# Machine Learning Approaches for the Prediction of Hepatitis B and C Seropositivity

**DOI:** 10.3390/ijerph20032380

**Published:** 2023-01-29

**Authors:** Valeriu Harabor, Raluca Mogos, Aurel Nechita, Ana-Maria Adam, Gigi Adam, Alina-Sinziana Melinte-Popescu, Marian Melinte-Popescu, Mariana Stuparu-Cretu, Ingrid-Andrada Vasilache, Elena Mihalceanu, Alexandru Carauleanu, Anca Bivoleanu, Anamaria Harabor

**Affiliations:** 1 Clinical and Surgical Department, Faculty of Medicine and Pharmacy, ‘Dunarea de Jos’ University, 800216 Galati, Romania; 2 Department of Mother and Child, ‘Grigore T. Popa’ University of Medicine and Pharmacy, 700115 Iasi, Romania; 3 Department of Pharmaceutical Sciences, Faculty of Medicine and Pharmacy, ‘Dunarea de Jos’ University, 800216 Galati, Romania; 4 Department of Mother and Newborn Care, Faculty of Medicine and Biological Sciences, ‘Ștefan cel Mare’ University, 720229 Suceava, Romania; 5 Department of Internal Medicine, Faculty of Medicine and Biological Sciences, ‘Ștefan cel Mare’ University, 720229 Suceava, Romania; 6 Medical Department, Faculty of Medicine and Pharmacy, ‘Dunarea de Jos’ University, 800216 Galati, Romania

**Keywords:** machine learning, hepatitis B, hepatitis C, screening, prediction

## Abstract

(1) Background: The identification of patients at risk for hepatitis B and C viral infection is a challenge for the clinicians and public health specialists. The aim of this study was to evaluate and compare the predictive performances of four machine learning-based models for the prediction of HBV and HCV status. (2) Methods: This prospective cohort screening study evaluated adults from the North-Eastern and South-Eastern regions of Romania between January 2022 and November 2022 who underwent viral hepatitis screening in their family physician’s offices. The patients’ clinical characteristics were extracted from a structured survey and were included in four machine learning-based models: support vector machine (SVM), random forest (RF), naïve Bayes (NB), and K nearest neighbors (KNN), and their predictive performance was assessed. (3) Results: All evaluated models performed better when used to predict HCV status. The highest predictive performance was achieved by KNN algorithm (accuracy: 98.1%), followed by SVM and RF with equal accuracies (97.6%) and NB (95.7%). The predictive performance of these models was modest for HBV status, with accuracies ranging from 78.2% to 97.6%. (4) Conclusions: The machine learning-based models could be useful tools for HCV infection prediction and for the risk stratification process of adult patients who undergo a viral hepatitis screening program.

## 1. Introduction

Hepatitis B infection is caused by the hepatitis B Virus (HBV), a deoxyribonucleic acid (DNA) virus belonging to the Hepadnaviridae family and the Orthohepadnavirus genus [1]. It is transmitted through contact with contaminated blood or bodily fluids, most frequently through intravenous drug use, sexual contact, or vertical transmission from mother to child [1]. HBV prevalence is decreasing in the developed countries due to vaccination, but it remains high in endemic areas due to vertical transmission [2,3]. The primary determinant of the hepatitis course is the age of HBV infection, so that the vast majority of perinatally infected individuals acquire chronic hepatitis B [4].

According to a systematic analysis, in 2019, the estimated global, all-age prevalence of chronic HBV infection was 4.1%, corresponding to 316 million infected people [5]. Moreover, there was a 31.3% decline in all-age prevalence between 1990 and 2019, with a more marked decline of 76.8% in prevalence in children younger than 5 years. The World Health Assembly launched the WHO Global Health Sector Strategy on Viral Hepatitis (WHO-GHSS) in 2016, with the goal of eradicating viral hepatitis as a public health concern [6]. The WHO-GHSS proposed impact targets of 30% fewer new hepatitis B cases and 10% fewer HBV-related deaths by 2020, and 95% fewer new cases and 65% fewer deaths by 2030, compared to the baseline year of 2015.

The hepatitis C virus (HCV), a single-stranded RNA virus, causes hepatitis C infection. HCV is a Flaviviridae family and Hepacivirus genus virus that is primarily spread through direct bloodstream inoculation [7]. According to recent studies, there are approximately 71 million persons infected with HCV, which amounts to a global prevalence of 1.0% [8]. The WHO-GHSS aim to reduce the HCV incidence by 80%, and the HCV-related mortality by 65% [6].

The identification of infected people is the first step in the sequence of care, and this can be achieved with a systematic screening program, especially for patients at risk. All persons who are seronegative should receive hepatitis B vaccine [9]. Persons who are at risk for HBV/HCV infection are represented by newborns from infected mothers, hemodialysis patients, individuals infected with human immunodeficiency virus (HIV), drug users, migrants from countries with high HBV/HCV prevalence rates, prisoners, people who have received blood products, war veterans, and people with risky sexual behavior [10,11,12].

The risk profile identification is essential for a proper selection of target population that will be further screened. Moreover, the governmental agencies need to constantly adapt their strategies in order to offer the best screening opportunities that are also cost-effective [13]. Therefore, it is important to evaluate the performances of the current screening programs and to constantly improve their quality.

Artificial intelligence and machine learning approaches have the ability to outperform traditional hepatitis screening strategies and to evaluate large datasets in order to provide a full picture of regional epidemiological profiles. The machine learning-based methods for disease prediction include random forest (RF), decision trees (DT), gradient boosting (GB), naïve Bayes (NB), and support vector machine (SVM) [14]. Until now, machine learning-based methods have been employed for the prediction of the type and duration of antiviral therapy [15,16], stage of HCV infection [17], the occurrence of hepatic fibrosis and hepatocellular carcinoma related to hepatitis infection [18,19], and for classification purposes [20].

A recent study used three neural networks for the prediction of HBV and HCV incidence in a cohort from China, using surveillance data from a 13-year time-frame [21]. The results showed that the Long Short-Term Memory (LSTM) prediction model, Recurrent Neural Network (RNN) model, and the Back Propagation Neural Network (BPNN) model had significant predictive performance for the early detection of the disease incidents.

Although the literature supports the use of these approaches for disease prediction, data are heterogeneously reported, and few studies concentrate on the patient’s epidemiological profile. The aim of this study was to evaluate and compare the predictive performances of four machine-learning based models for the prediction of HBV and HCV seropositivity.

## 2. Materials and Methods

We conducted a prospective cohort screening study of adult persons from the northeastern and southeastern regions of Romania, between January 2022 and November 2022 (LIVE(RO) 2-EST). Ethical approval for this study was obtained from the Institutional Ethics Committee of University of Medicine and Pharmacy ‘Grigore T. Popa’ (No. 151/13 January 2022). Informed consent was obtained from all participants included in the study. All methods were carried out in accordance with relevant guidelines and regulations.

We recruited participants at the time of the routine family physician evaluation. The inclusion criteria taken into consideration were age of ≥18 and a home address located in the northeastern and southeastern regions of Romania. Exclusion criteria comprised pregnant patients, arrested persons, incomplete medical records, or those who were unable to offer informed consent.

A structured questionnaire was applied for all participants by family physician, and the following data were recorded for the purpose of this study: demographic data, age, level of education, ethnicity, employment status, vulnerability due to medium, ethnicity, work or other situations, HBV vaccinal status, previous diagnosis and/or treatment for viral hepatitis, contact with hepatitis viruses in family, through sexual contact, work, or other instances, previous blood transfusions, hemodialysis, surgery, hospitalization, oral procedures, work or house-related accidents that necessitated hospitalization, cuts/other injuries with sharp objects, incarceration, tattoos/piercings, use of intravenous drugs, one or more unprotected sexual intercourse(s) with one or multiple partners, previous sexually transmitted infections.

All patients underwent rapid blood testing for HBs antigens and for HCV antibodies using immunochromatographic tests, and after the results came back, the patients were asked whether or not they would like further referring to a diagnostic center from the country.

A total of 1359 patients were included in the preliminary analysis of this study, and were divided into three groups: those who had HBV infection (116 patients, group 1), those who had HCV infection (116 patients, group 2), and a control group, without infection (116 patients, group 2). HBV-HCV coinfection was identified in two patients who were excluded from the analysis due to small sample size.

In the first stage of the statistical analysis, each variable was evaluated with chi-squared and Fisher’s exact tests for categorical variables, which were presented as frequencies with corresponding percentages, and *t*-tests for continuous variables, which were presented as means and standard deviations (SD).

Multinomial logistic regression was used to determine whether or not there is a statistically significant difference between the groups regarding their clinical characteristics derived from the standardized questionnaire. The statistical analyses were performed using STATA SE (version 17, 2021, StataCorp LLC, College Station, TX, USA).

In the second stage of the analysis, we evaluated the predictive performance of four machine learning-based models: support vector machine, naïve Bayes, random forest algorithm, and K nearest neighbors (KNN).

A SVM is a supervised learning algorithm used for classification and regression [22,23]. This algorithm is a relatively new method that has shown promising results in recent years for disease prediction. SVM classifiers are based on linear classifiers and seek to select a line that is slightly more confident.

NB is a classification technique based on the Bayes’ theorem [24]. This theorem can predict the likelihood of an occurrence depending on prior knowledge of the event conditions. This classifier asserts that a given characteristic in a class is not directly related to any other feature, despite the fact that features in that class may be interdependent [25].

Random forests are ensemble classifiers that randomly learn multiple decision trees [26]. The random forest approach consists of a training stage in which many decision trees are built and a testing step in which an outcome variable is classified or predicted based on an input vector [25]. The different decision trees of a RF are trained using the different parts of the training dataset. To classify or predict a new sample, the input vector of that sample is needed to pass down with each DT of the forest. Each DT then considers a different part of that input vector and offers a prediction outcome. The forest then selects the prediction with the greatest number of ‘votes’ (for discrete outcomes) or the average of all trees in the forest (for numeric outcomes).

KNN is a supervised machine-learning algorithm predominantly used for classification and prediction purposes [25]. It is able to classify datasets using a training model similar to the testing query by taking into account the K nearest training data points (neighbors) which are the closest to the query it is testing [27]. The algorithm performs a majority voting rule to check which classification to finalize [28].

The data were segregated into data for testing (70%) and training (30%). In order to protect the results from overfitting, all models underwent a 5-fold cross validation. Their true positive rates (TPR), false negative rates (FNR), positive predictive values (PPV), false detection rates (FDR), accuracies, values for area under the curve (AUC), precision, recall, and F1 scores were calculated, and compared for HBV and HCV seropositivity version. The models were constructed and analyzed using Matlab (version R2021b, The MathWorks, Inc., Natick, MA, USA).

## 3. Results

A total of 1359 participants were evaluated in our prospective study. Their demographic characteristics are presented in Table 1, segregated into the following groups: patients with HBV (38 patients, group 1), patients with HCV (group 2, 33 patients), and controls (group 3, 1288 patients). Significantly more widowed (*p* < 0.001), employed (*p* = 0.01), and agricultural workers (*p* < 0.001) were identified in the first group, while the second group comprised persons who were older (*p* < 0.001), females (*p* = 0.01), with an educational background predominantly in the ISCED 1-3 interval (*p* = 0.02), and widowed (*p* < 0.001) compared to control.

The questionnaire results for the main groups are presented in Table 2. Both HBV and HCV patients reported a significantly higher personal or family history of viral hepatitis compared with control (*p* < 0.001). Moreover, both groups had significantly higher proportion of risky professions, hospitalizations, hemodialysis, surgeries, and dental procedures (*p* < 0.05). Apart from that, the first group had significantly more severe accidents (*p* = 0.002) and blood transfusions (*p* < 0.001) than the control group.

In the second stage of the analysis, we incorporated the patients’ clinical characteristics into four machine learning-based models, and we calculated their predictive performance (Table 3). KNN achieved the highest accuracy when predicting HCV status (98.1%), with an AUC value of 0.67. The SVM had equal accuracies (97.6%) for the prediction of both HBV and HCV status, but the AUC value was higher for HCV classification (0.89 versus 0.80). Both RF and NB performed best when used to predict the HCV status (RF: accuracy—97.6%; AUC—0.79; NB: accuracy—95.7%; AUC—0.85). In terms of sensitivity, it was higher for algorithms that predicted HCV status, with KNN having the highest sensitivity (100%).

## 4. Discussion

This is the first prospective study in the literature that trained four machine learning-based models (SVM, RF, NB, and KNN) for the prediction of hepatitis B and C seropositivity in a cohort of adult patients from Romania using clinical parameters determined during family physicians’ visits.

Our results showed that all evaluated models performed better when used to predict HCV status. The highest predictive performance was achieved by KNN algorithm (accuracy: 98.1%), followed by SVM and RF with equal accuracies (97.6%) and NB (95.7%). The sensitivity was modest for HBV status prediction, only one model (SVM) achieving a sensitivity of 40%.

SVM increases class separation and reduces expected prediction error and is applicable for the analysis of high-dimensionality data with small sample size [29,30,31]. The bagging algorithm serves as the foundation for RF, which employs ensemble learning [32]. It creates as many trees on the subset of the data and combines the output of all the trees. In doing so, it lessens the issue of overfitting in decision trees, as well as lowers variance and raises accuracy. On the other hand, NB is suitable for solving multi-class prediction problems, especially when using small datasets, and has much lower costs than RF [33]. Finally, one of the biggest advantages of KNN model is that it can be used both for classification and regression problems, but does not perform well on imbalanced data [34].

A recent case-control study by Majzoobi et al. evaluated the predictive performance of four ensemble learning methods (bagging, AdaBoost, RF, and logistic regression) for the prediction of HBV and HCV infection [35]. The authors demonstrated superior predictive performances of RF when used to predict both HBV (accuracy: 66%) and HCV infection (accuracy: 77%) compared to the other models. Although we obtained better results in terms of accuracies for HBV (accuracy: 78.5%) and HCV infection (accuracy: 97.6%), the model was outperformed by KNN.

Another recent study by Zhou et al, used three machine learning methods (RF, KNN, and SVM) for analyzing correlations among chronic hepatitis B inflammation grades, gene expressions and clinical parameters (serum alanine amino transaminase, aspartate amino transaminase, and HBV-DNA), and for predicting inflammation grades by using clinical parameters and/or gene expressions. The authors showed that KNN had the highest accuracy (76.6%) compared to SVM (accuracy: 65.4%) and RF (accuracy 72.8%) when using all the evaluated types of data [36].

Chen et al. evaluated the predictive performance of four classifiers (SVM, NB, RF, and KNN) in order to build a decision-support system that would improve the hepatitis B staging using real-time elastography data. The results indicated that RF had the highest accuracy for the prediction of stage 0 (82.8%), 1 (81.1%), 2 (88%), and 3 (91.2%) of liver fibrosis [37].

These studies outline the high predictive performance of machine learning-based models in various settings and use multiple types of data. However, the costs of performing such analyses are high, especially for gene sequencing. In order to provide the best screening strategy, it is important to identify the subjects with the highest risk based on data that can be obtained with minimal costs and thus constitute an advantage of our study that indicates a good predictive performance of machine learning models using data which can be easily obtained.

Our study has several limitations, including a small cohort of patients and number of predictors. At the same time, the trained models have the advantage of an easier implementation by the physicians. All chosen machine learning-based models have the ability to handle small sample data [38]. Moreover, the used algorithms have proven superior predictive performance when applied for datasets based mainly on categorical predictors in comparison with other models such as gradient boosting [39], artificial neural networks [40], support vector machines, extreme gradient boosting, multilayer perceptron [41] or linear discriminant analysis [42].

We hypothesize that the model accuracies could be improved by adding repeated serum measurements and liver elastography parameters which have been proven to be useful biomarkers for viral hepatitis prediction [43,44,45,46].

Further studies on larger cohorts of patients could evaluate the predictive performance of these ML-based models in different settings and populations. The results could aid clinicians in the risk stratification process of adult patients who undergo a screening program, and could help optimize the costs of the screening programs.

## 5. Conclusions

The machine learning-based models could be useful tools for HCV infection prediction and for the risk stratification process of adult patients who undergo a viral hepatitis screening program.

The results for HBV prediction using only clinical characteristics are modest in terms of predictive performance.

These findings are important for clinicians and public health specialists because they can be further validated and incorporated into national screening programs in order to optimize them and to reduce their costs.

## Figures and Tables

**Table 1:** Demographic characteristics of the patients included in the main groups.

Patient’s Characteristics	Group 1 (HVB, *n* = 38)	Group 3 (Control, *n* = 1288)	*p* Value	Group 2 (HVC, *n* = 33)	Group 3 (Control, *n* = 1288)	*p* Value
Age, years (mean ± SD)	56.05 ± 2.13	57.71 ± 0.47	0.56	75.54 ± 1.84	57.71 ± 0.47	<0.001
Medium (*n*/%)	Urban = 9 (23.68%)	Urban = 906 (66.81%)	0.14	Urban = 11 (33.33%)	Urban = 448 (32.92%)	0.96
	Rural = 29 (76.32 %)	Rural = 450 (33.19%)		Rural = 22 (66.67%)	Rural = 913 (67.08%)	
Gender (*n*/%)	Male = 18 (47.37%)	Male = 850 (62.68%)	0.13	Male = 6 (18.18%)	Male = 518 (38.06%)	0.01
	Female = 20 (52.63%)	Female = 506 (37.32%)		Female = 27 (81.82%)	Female = 843 (61.94%)	
Level of education (*n*/%)	ISCED 1 = 1 (2.63%)	ISCED 1 = 10 (0.74%)	0.21	ISCED 1 = 1 (3.03%)	ISCED 1 = 10 (0.74%)	0.02
	ISCED 2 = 12 (31.58%)	ISCED 2 = 438 (32.30%)		ISCED 2 = 6 (18.18%)	ISCED 2= 444 (32.62%)	
	ISCED 3 = 14 (36.84%)	ISCED 3 = 371 (27.36%)		ISCED 3 = 15 (45.45%)	ISCED 3 = 370 (27.19%)	
	ISCED 4 = 9 (23.68%)	ISCED 4 = 472 (34.81%)		ISCED 4 = 8 (24.24%)	ISCED 4 = 473 (34.75%)	
	ISCED 5 = 1 (2.63%)	ISCED 5 = 15 (1.11%)		ISCED 5 = 0 (0%)	ISCED 5 = 16 (1.18%)	
	ISCED 6 = 1 (2.63%)	ISCED 6 = 50 (3.69%)		ISCED 6 = 13 (9.09%)	ISCED 6 = 48 (3.53%)	
Marital status (*n*/%)	Married = 27 (71.05%)	Married = 1042 (76.84%)	<0.001	Married = 14 (42.42%)	Married = 1042 (76.84%)	<0.001
	Unmarried = 4 (10.53%)	Unmarried = 268 (19.76%)		Unmarried = 1 (3.03%)	Unmarried = 268 (19.76%)	
	Widowed = 5 (13.16%)	Widowed = 35 (2.58%)		Widowed = 16 (48.48%)	Widowed = 35 (2.58%)	
	Divorced = 1 (2.63%)	Divorced = 0 (0%)		Divorced = 0 (0%)	Divorced = 0 (0%)	
	Others = 0 (0%)	Others = 9 (0.66 %)		Others = 0 (0%)	Others = 9 (0.66 %)	
	Undeclared = 1 (2.63%)	Undeclared = 2 (0.15%)		Undeclared = 1 (2.63%)	Undeclared = 2 (0.15%)	
Employment status (*n*/%)	Self-employed = 3 (7.89%)	Self-employed = 18 (1.32%)	0.01	Self-employed = 2 (6.06%)	Self-employed = 18 (1.32%)	0.10
	Employed = 10 (26.32%)	Employed = 3 (9.09%)		Employed = 4 (10.53%)	Employed = 3 (9.09%)	
	Unemployed = 0 (0%)	Unemployed = 11 (0.81%)		Unemployed = 0 (0%)	Unemployed = 11 (0.81%)	
	Inactive = 25 (65.79%)	Inactive = 1089 (80.01%)		Inactive = 28 (84.85%)	Inactive = 1089 (80.01%)	
Vulnerability due to work (*n*/%)	Agricultural worker = 3 (7.89%)	Agricultural worker = 11 (0.81%)	<0.001	Agricultural worker = 0 (0%)	Agricultural worker = 3 (7.89%)	0.003
	Unassured = 3 (7.89%)	Unassured = 200 (14.75%)		Unassured = 0 (0%)	Unassured = 3 (7.89%)	
Vulnerability due to special situations/%)	Single parents = 4 (10.53%)	Single parents = 32 (2.36%)	0.18	Single parents = 0 (0%)	Single parents = 32 (2.36%)	0.06
	Previous foster care = 0 (0%)	Previous foster care = 24 (1.77%)		Previous foster care = 2 (6.06%)	Previous foster care = 24 (1.77%)	
	Addicts = 0 (0%)	Addicts = 3 (0.22%)		Addicts = 0 (0%)	Addicts = 3 (0.22%)	
	Domestic violence victims = 0 (0%)	Domestic violence victims = 14 (1.03%)		Domestic violence victims = 0 (0%)	Domestic violence victims = 14 (1.03%)	
	Human trafficking victims = 0 (0%)	Human trafficking victims = 11 (0.81%)		Human trafficking victims = 0 (0%)	Human trafficking victims = 11 (0.81%)	
	Minimal wage = 9 (23.68%)	Minimal wage = 265 (19.54%)		Minimal wage = 1 (3.03%)	Minimal wage = 265 (19.54%)	
	Disabled = 1 (2.63%)	Disabled = 25 (21.84%)		Disabled = 1 (3.03%)	Disabled = 25 (21.84%)	

HBV—hepatitis B virus; HCV—hepatitis C virus; SD—standard deviation; ISCED—International Standard Classification of Education.

**Table 2:** Clinical characteristics of the patients included in the main groups.

Question Resume	Group 1 (HBV, *n* = 38)	Group 3 (Control, *n* = 1288)	*p* Value	Group 2 (HCV, *n* = 33)	Group 3 (Control, *n* = 1288)	*p* Value
Vaccinal status for HBV (*n*/%)	Yes = 2 (5.26%)	Yes = 23 (1.70%)	0.14	Yes = 0 (0%)	Yes = 23 (1.70%)	0.54
Previous diagnosis of hepatitis (*n*/%)	Yes = 3 (7.89 %)	Yes = 13 (0.96 %)	0.008	Yes = 10 (30.30%)	Yes = 13 (0.96 %)	<0.001
Type of hepatitis (*n*/%)	HBV = 1 (3.03%)	HBV = 0 (0%)	<0.001	HBV = 3 (7.89%)	HBV = 0 (0%)	0.01
	HCV = 9 (27.27%)	HCV = 0 (0%)		HCV = 0 (0%)	HCV = 0 (0%)	
Family member diagnosed with viral hepatitis (*n*/%)	Yes = 11 (28.95%)	Yes = 2 (1.92%)	<0.001	Yes = 6 (18.18%)	Yes = 2 (1.92%)	<0.001
Sexual partner diagnosed with viral hepatitis (*n*/%)	Yes = 8 (21.05%)	Yes = 31 (2.29%)	<0.001	Yes = 4 (12.12%)	Yes = 31 (2.29%)	0.003
Profession that involves contact with other people’s blood (*n*/%)	Yes = 4 (10.53%)	Yes = 10 (0.74%)	<0.001	Yes = 2 (6.06%)	Yes = 10 (0.74%)	0.04
Previous blood transfusions (*n*/%)	Yes = 3 (7.89%)	Yes = 3 (0.22%)	<0.001	Yes = 2 (6.06%)	Yes = 3 (0.22%)	0.008
Previous hemodialysis (*n*/%)	Yes = 2 (5.26%)	Yes = 7 (0.52%)	0.02	Yes = 3 (9.09%)	Yes = 7 (0.52%)	0.001
Previous surgery (*n*/%)	Yes = 16 (42.11%)	Yes = 93 (6.86%)	<0.001	Yes = 26 (78.79%)	Yes = 93 (6.86%)	<0.001
Previous hospitalization (*n*/%)	Yes = 22 (57.89%)	Yes = 103 (7.60%)	<0.001	Yes = 26 (78.79%)	Yes = 93 (6.86%)	<0.001
Previous dental procedures (*n*/%)	Yes = 15 (39.47%)	Yes = 183 (6.12%)	<0.001	Yes = 25 (75.76%)	Yes = 183 (6.12%)	<0.001
Previous accidents (*n*/%)	Yes = 2 (5.26%)	Yes = 1(0.07%)	0.002	Yes = 1 (3.03%)	Yes = 1(0.07%)	0.06
Previous incarceration (*n*/%)	Yes = 1 (2.63 %)	Yes = 2 (0.15%)	0.08	Yes = 2 (6.06 %)	Yes = 2 (0.15%)	0.002
Tattoos/piercings (*n*/%)	Yes = 7 (18.42%)	Yes = 137 (10.10%)	0.08	Yes = 12 (36.36%)	Yes = 137 (10.10%)	<0.001
Use of intravenous drugs (*n*/%)	Yes = 0 (0%)	Yes = 2 (0.15%)	0.94	Yes = 1 (3.03%)	Yes = 2 (0.15%)	0.04
Unprotected sexual contact (*n*/%)	Yes = 2 (5.26 %)	Yes = 1 (0.07%)	0.002	Yes = 0 (0%)	Yes = 1 (0.07%)	0.7
Tattoos/piercings (*n*/%)	Yes = 1 (2.63%)	Yes = 12 (0.88%)	0.17	Yes = 0 (0%)	Yes = 12 (0.88%)	0.7

HBV—hepatitis B virus; HCV—hepatitis C virus.

**Table 3:** The predictive performance of machine learning-based models for HVB and HVC prediction.

ML Model	Type of Hepatitis Virus	Se (%)	Sp (%)	FPR (%)	FDR (%)	Accuracy (%)	AUC Value	Precision	Recall	F1 Score	Gini
SVM	HBV	40	98.3	1	77.7	97.6	0.80	0.22	0.40	0.29	0.60
	HCV	66.6	97.8	2	81.8	97.6	0.89	0.18	0.67	0.29	0.78
RF	HBV	8	99.6	0.3	11	78.5	0.87	0.89	0.08	0.15	0.75
	HCV	55.5	98.5	1	54.5	97.6	0.79	0.45	0.56	0.50	0.59
NB	HBV	7	99.3	0.6	22.2	78.3	0.78	0.71	0.78	0.88	0.58
	HCV	23	98	1	72.7	95.7	0.85	0.27	0.23	0.25	0.70
KNN	HBV	11.8	98.7	1	25	78.2	0.70	0.75	0.12	0.21	0.40
	HCV	100	98.1	1	72.7	98.1	0.67	0.27	1	0.43	0.35

HVB—hepatitis B virus; HVC—hepatitis C virus; ML—machine learning; KNN—decision trees; NB—naïve Bayes; SVM—support vector machine; RF—random forest; Se—sensitivity; Sp—specificity; FPR—false positive rate; FDR—false detection rate; AUC—area under the curve.

## Data Availability

The data presented in this study are available on request from the corresponding author. The data are not publicly available due to local policies.
